# Multicentre study of the burden of multidrug-resistant bacteria in the aetiology of infected diabetic foot ulcers

**DOI:** 10.4102/ajlm.v10i1.1261

**Published:** 2021-03-23

**Authors:** Adeyemi T. Adeyemo, Babatope Kolawole, Vincent O. Rotimi, Aaron O. Aboderin

**Affiliations:** 1 Department of Medical Microbiology and Parasitology, Obafemi Awolowo University Teaching Hospitals, Ile-Ife, Nigeria; 2 Department of Medicine, Faculty of Clinical Science, Obafemi Awolowo University, Ile-Ife, Nigeria; 3 Department of Microbiology, Faculty of Medicine, Kuwait University, Kuwait City, Kuwait; 4 Department of Medical Microbiology and Parasitology, Faculty of Basic Medical Science, Obafemi Awolowo University, Ile-Ife, Nigeria

**Keywords:** infection, diabetic foot, ulcers, multidrug-resistance, bacteria, antibiotic, anaerobic culture, samples

## Abstract

**Background:**

Infected diabetic foot ulcer (IDFU) is a public health issue and the leading cause of non-traumatic limb amputation. Very few published data on IDFU exist in most West African countries.

**Objective:**

The study investigated the aetiology and antibacterial drug resistance burden of IDFU in tertiary hospitals in Osun state, Nigeria, between July 2016 and April 2017.

**Methods:**

Isolates were cultured from tissue biopsies or aspirates collected from patients with IDFU. Bacterial identification, antibiotic susceptibility testing and phenotypic detection of extended-spectrum beta-lactamase and carbapenemase production were done by established protocols. Specific resistance genes were detected by polymerase chain reaction.

**Results:**

There were 218 microorganisms isolated from 93 IDFUs, comprising 129 (59.2%) Gram-negative bacilli (GNB), 59 (27.1%) Gram-positive cocci and 29 (13.3%) anaerobic bacteria. The top five facultative anaerobic bacteria isolated were: *Staphylococcus aureus* (34; 15.6%), *Escherichia coli* (23; 10.6%), *Pseudomonas aeruginosa* (20; 9.2%), *Klebsiella pneumoniae* (19; 8.7%) and *Citrobacter* spp. (19; 8.7%). The most common anaerobes were *Bacteroides* spp. (7; 3.2%) and *Peptostreptococcus anaerobius* (6; 2.8%). Seventy-four IDFUs (80%) were infected by multidrug-resistant bacteria, predominantly methicillin-resistant *S. aureus* and GNB producing extended-spectrum β-lactamases, mainly of the CTX-M variety. Only 4 (3.1%) GNB produced carbapenemases encoded predominantly by *bla*_VIM_. Factors associated with presence of multidrug-resistant bacteria were peripheral neuropathy (adjusted odds ratio [AOR] = 4.05, *p* = 0.04) and duration of foot infection of more than 1 month (AOR = 7.63, *p* = 0.02).

**Conclusion:**

Multidrug-resistant facultative anaerobic bacteria are overrepresented as agents of IDFU. A relatively low proportion of the aetiological agents were anaerobic bacteria.

## Introduction

Infected diabetic foot ulcer (IDFU) is associated with inlammation or purulence occurring in sites below the ankle in persons with diabetes mellitus.^1^ It is a major global public health issue with a substantial medical, socio-economic and psychological burden. Infected diabetic foot ulcer is one of the most common diabetes-related infections in clinical practice, and a common indication for hospital admission.^1^ Ulceration often precedes foot infection in diabetic patients, with peripheral vascular disease, peripheral neuropathy and visual impairment and immunological disturbances also playing contributory roles. Infection impairs the healing process and aggravates the condition of patients with diabetic foot ulcer (DFU) and could lead to great disability, septicaemia and death if not promptly and properly managed. At 7.2% (95% confidence interval [CI]: 5.1–9.3%) and higher than the global prevalence of 6.3% (95% CI: 5.4–7.3%), Africa has the second highest global prevalence of DFU, a precursor of IDFU.^2^ Foot infections are more common and lethal in Africa than elsewhere globally.^3^ Between 25% and 60% of diabetic patients with a background foot ulcer will develop IDFU which remains a major reason for non-traumatic amputation of the lower limbs.^4^ The foot infection can progress to irreversible septic gangrene which often necessitates life-saving amputation of the lower limb.^5^ Patients with IDFU have 15–46 times higher risk of limb amputation than those with non-diabetic related ulcers.^6^ More than 1 million diabetic patients may require limb amputation worldwide yearly, and a greater percentage of them are in developing countries.^7^

Wide varieties of organisms, including anaerobic bacteria, have been implicated in the aetiology of IDFU depending on the severity of infection and time from onset to presentation at healthcare facilities. Advanced IDFUs with features of sepsis at admission usually harbour anaerobic pathogens.^8^ The emergence and current global threat of antimicrobial resistance in the face of dwindling antibiotics in the development pipeline has added a new twist to the burden of IDFU.^9^ Increasing involvement of multidrug-resistant organisms (organisms resistant to at least three different antimicrobial classes)^10^ in diabetic patients with infected foot ulcers has significantly reduced antibiotic treatment options, thus posing a serious challenge particularly in resource-constrained low- and middle-income countries where access to antimicrobial drugs is of grave concern.^11^ It has also increased the length of hospital stay and cost of treatment, and caused additional morbidity and mortality.^12^ These situations have assumed worrisome trends in which resistance is building up to antibiotics of last resort; pathogens showing considerable resistance to vancomycin and carbapenems are particularly becoming more common as agents of foot infection in diabetic patients.^13^ Various studies have reported many independent risk factors and predictors of multidrug-resistant IDFU including previous hospitalisation for the same wound, prolonged antibiotic therapy, ulcer type and increased ulcer size, presence of osteomyelitis, poor glycaemic control, prolonged duration of foot ulcer infection as well as proliferative retinopathy.^14,15,16,17,18^ According to Bakele et al., predictors of lower limb amputation by multivariate logistic regression analysis were advanced ulcer grade, inappropriate antibiotic use, overweight, obesity, poor blood glucose control and neuropathy.^19^ Furthermore, albuminuria, diabetic nephropathy and Charcot arthropathy were noted as predictors of poor healing of diabetic foot ulcer.^20^

A recent systematic review and meta-analysis on the global burden of diabetic foot ulceration in Cameroon, West Africa, concluded that paucity of data impedes strategies for treatment and prevention of foot infections in diabetic patients.^2^ Thus, our study was designed to determine the prevalent bacteria involved in IDFUs, assess the burden of multidrug-resistance (MDR) among the isolates and evaluate the associated risk factors.

## Methods

### Ethical considerations

Ethical approval for this study was granted by the Ethics and Research Committees of the Obafemi Awolowo University Teaching Hospitals Complex and Ladoke Akintola University College of Technology with protocol numbers ERC/2015/11/02 and LTH/ER/2016/01/254. Information about the study and participant involvement was fully explained to patients, and properly signed and dated written informed consent forms were obtained from patients before their recruitment into the study. Results of wound biopsy microscopy, culture and sensitivity were made available for patients’ management.

### Study population

The prospective, cross-sectional, multicentre, hospital-based study was carried out in Osun state, southwest Nigeria, between July 2016 and April 2017. It included three existing tertiary healthcare facilities in the state: Obafemi Awolowo University Teaching Hospitals Complex, Wesley Guild Hospital, Ilesa, and Ladoke Akintola University of Technology Teaching Hospital, Osogbo. All consecutive diabetic patients (both hospitalised and outpatients) with foot infections meeting the criteria for diagnosis of IDFU seen and managed at these hospitals were recruited into the study. They were clinically assessed and foot lesions graded according to the diabetic foot infection severity classification system issued by the Infectious Disease Society of America.^8^ Only non-duplicate patients and samples were included in the study. All inpatients were followed up with regular check-ups physically in the wards until they either died or were discharged.

### Sample collection and bacterial identification

Aspirates were obtained from deep-seated abscesses, and tissue samples were collected after washing the wound vigorously with sterile saline and debridement of the slough to exclude mere colonisers. Necrotic tissues were curetted into Anaerobic Basal Broth (Oxoid, Basingstoke, Hants, United Kingdom) for anaerobic culture. The samples were immediately transported to the laboratory and processed within 2 h of sample collection by inoculating them onto a set of selective and non-selective media which were: 5% (volume/volume) sheep blood agar (BA; Oxoid, Basingstoke, Hants, United Kingdom), MacConkey agar (Oxoid, Basingstoke, Hants, United Kingdom), chocolate agar and anaerobic basal agar (Oxoid, Basingstoke, Hants, United Kingdom) supplemented with 5% (abscesses) laked sheep blood, Vitamin K1 (1 *µ*g/mL), L-cysteine hydrochloride (5 *µ*g/mL) and gentamicin (100 *µ*g/mL) (gentamicin blood agar).

Inoculated plain BA and MacConkey agar plates were incubated in air and chocolate agar plates in CO_2_ at 37 °C for 24 h. Inoculated plain gentamicin BA plates, as well as gentamicin BA with kanamycin (75 *µ*g/L) and vancomycin (5 *µ*g/L) supplements, were incubated under anaerobic conditions made up of 80% H_2_, 10% CO_2_ and 10% N_2_ for 48 h and extended for 5 days if necessary; anaerobiosis was achieved using a Bactron anaerobic chamber (Sheldon Manufacturing, Inc., Cornelius, Oregon, United States). Representative colonies were identified by colonial morphology, Gram staining characteristics and conventional biochemical tests including catalase and oxidase tests. Facultative anaerobic Gram-negative bacilli (GNB) and *Streptococcus* spp. were further identified with Microbact^™^ GNB 24E (Oxoid, Basingstoke, Hants, United Kingdom) and RapID^™^ STR (Thermo Fisher Scientific, Remel Products, Lexena, Kansas, United States), while *Staphylococcus* spp. were further identified with a coagulase test, characteristic growth appearance on mannitol salt agar and a DNAse test. The obligate anaerobes were identified by RapID^™^ ANA II (Thermo Fisher Scientific, Remel Products, Lexena, Kansas, United States). Yeast isolates were identified by Gram staining and germ tube tests. Quality control strains, *Staphylococcus aureus* ATCC 25923, *Escherichia coli* ATCC 25922, *Bacteroides fragilis* ATCC 25285 and *Peptostreptococcus anaerobius* ATCC 27337, were used to assess the quality of the media and identification systems. The quality of our bacterial identification system and procedures (for aerobes, facultative anaerobes and obligate anaerobes) were assured by ensuring that the control bacterial strains were identified to their names.

### Antibiotic susceptibility test

Antibiotic susceptibility testing for aerobes and facultative anaerobes was performed using the modified Kirby-Bauer disc diffusion technique as recommended by the Clinical and Laboratory Standards Institute (CLSI).^21^ Gram-positive isolates were tested with the following antibiotic discs (Oxoid, Basingstoke, Hants, United Kingdom): penicillin (10 *µ*g), cefuroxime (30 *µ*g), ceftriaxone (30 *µ*g), cefepime (30 *µ*g), co-amoxiclav (20/10 *µ*g), ciprofloxacin (5 *µ*g), chloramphenicol (30 *µ*g), gentamicin (10 *µ*g), amikacin (10 *µ*g), trimethoprim-sulfamethoxazole (1.25 *µ*g/23.75 *µ*g), cefoxitin (30 *µ*g), erythromycin (15 *µ*g), ampicillin-sulbactam (10 *µ*g/10 *µ*g), piperacillin-tazobactam (100 *µ*g/10 *µ*g) and vancomycin (30 *µ*g). Vancomycin (256–0.015 *µ*g/mL) minimum inhibitory concentration (MIC) strip (Oxoid, Basingstoke, Hants, United Kingdom) was used to test for vancomycin resistance among methicillin-resistant *S. aureus* (MRSA). Gram-negative isolates were tested with the following antibiotic discs (Oxoid, Basingstoke, Hants, United Kingdom): ceftriaxone (30 *µ*g), ceftazidime (30 *µ*g), cefotaxime (30 *µ*g), cefepime (30 *µ*g), co-amoxiclav (20/10 *µ*g), ciprofloxacin (5 *µ*g), gentamicin (10 *µ*g), amikacin (10 *µ*g), chloramphenicol (30 *µ*g), aztreonam (30 *µ*g), trimethoprim-sulfamethoxazole (1.25 *µ*g/23.75 *µ*g), ampicillin-sulbactam (10 *µ*g/10 *µ*g), piperacillin-tazobactam (100 *µ*g/10 *µ*g), cefoxitin (30 *µ*g), meropenem (10 *µ*g) and ertapenem (10 *µ*g).

Discrete colonies were emulsified in sterile saline to match 0.5 McFarland turbidity standards from where confluence inocula were made on Mueller-Hinton agar (MHA) plates with sterile cotton swabs. The swabbed MHA plates were allowed to dry at room temperature and a set of six antibiotic discs were placed evenly spaced on each of the plates. Vancomycin resistance was tested in the methicillin-resistant *S. aureus* isolates by placing a MIC evaluation strip (Oxoid, Basingstoke, Hants, United Kingdom) on inoculated MHA. After 18–24 h of incubation, the diameter of the zone of inhibition around each antibiotic disc was measured and recorded. Vancomycin MIC values were also recorded for the *S. aureus.* Zones of inhibition of each antibiotic as well as vancomycin MIC values were interpreted as ‘sensitive’, ‘intermediate’ or ‘resistant’ in accordance with CLSI guidelines.^21^ Isolates with intermediate sensitivity were regarded as ‘resistant’. The quality of antibiotic susceptibility testing consumables (including antibiotic discs and MHA) and procedures were assured with bacterial control strains (*E. coli* ATCC 25922, *S. aureus* ATCC 25923 and *S. aureus* ATCC 43300). Zones of inhibition of tested antibiotics on the bacterial control strains fell within their quality control ranges according to CLSI.^21^

Extended-spectrum β-lactamase production was confirmed among Enterobacteriaceae and other GNB that showed reduced susceptibility to at least one of the tested third-generation cephalosporins (cefotaxime 30 *µ*g, ceftazidime 30 *µ*g and ceftriaxone 30 *µ*g) or aztreonam (30 *µ*g) by a combination disc diffusion test (CDDT).^21^ CDDT was done using both single discs of cefotaxime (30 *µ*g) and ceftazidime (30 *µ*g) and their respective clavulanic acid containing discs (cetotaxime-clavulanate 30/10 *µ*g, ceftazidime-clavulanate 30/10 *µ*g). A 5 mm or more increase in zone of inhibition of one or more combination discs as compared with their respective single discs was taken as confirmatory evidence of extended-spectrum beta-lactamase (ESBL) production.^21^

AmpC beta-lactamase production was detected by AmpC disc test as described by Anjali et al. on isolates which show resistance to at least one third-generation cephalosporin and a β-lactamase inhibitor.^22^ A broth culture of *E. coli* ATCC 25922 was adjusted to 0.5 McFarland turbidity standard and inoculated onto MHA plates. Sterile filter paper discs (6 mm) were moistened with distilled water (about 20 *µ*l) and up to five colonies of the test organism was transferred onto the filter paper. Afterwards, a cefoxitin (30 *µ*g) disc and the inoculated filter paper disc were placed next to each other and almost touching on inoculated media. This setup was incubated overnight at 37 °C. A flattening or indentation of the zone of inhibition of cefoxitin in the vicinity of the test disc (inoculated filter paper) indicated a phenotypic confirmatory evidence of AmpC β-lactamase production.^22^

Gram-negative bacilli with intermediate sensitivity or resistance to one or more carbapenems were tested for production of carbapenemases by the modified Hodge test and interpreted by CLSI guidelines.^21^ Organisms that were phenotypically MDR, including ESBL-producing GNB, carbapenem-resistant GNB and MRSA, were further tested for resistance-determining genes using polymerase chain reaction (PCR)-based protocols with specific oligonucleotide primers^23–27^ (Table 1); template bacterial DNA was extracted by the boiling method.^28^ Electrophoresis of each PCR product (5 *µ*L) was carried out in 1.5% (weight/volume) Agarose gel (Biomatik, Kitchener, Ontario, Canada) in 1X Tris-Acetate-EDTA (TAE) buffer for 45 min. The size of amplified products was estimated using 100 base pairs molecular weight marker (100–1200 base pairs).

**TABLE 1:** Oligonucleotides primers and amplification reactions for targeted resistance genes.

Target gene	Name	Primer sequence (5’ to 3’)	Amplicon size	Amplification reactions	References
*bla*_*CTX-M*_	CTX-M-F	TTGCGATGTGCAGTACCAGTAA	754 bp	Initial denaturation at 94 °C for 3 mins, followed by 35 cycles of denaturation at 94 °C for 45 s, annealing at 60 °C for 30 s and extension at 72 °C for 1 min, and a final extension at 72 °C for 3 mins	^23,24^
	CTX-M-R	CGAATATCGTTGGTGGTGCCATA	-		
*bla*_*SHV*_	SHV-F	ATTTGTCGCTTCTTTACTCGC	294 bp		
	SHV-R	TTTATGGCGTTACCTTTGACC	-		
*bla*_*TEM*_	TEM-F	ATGAGTATTCAACATTTCCGTG	404 bp		
	TEM-R	TTACCAATGCTTAATCAGTGAG	-		
*bla*_*KPC*_	KPC-F	ATGTCACTGTATCGCCGTCT	893 bp	Initial denaturation at 95 °C for 15 minutes, followed by 35 cycles of denaturation at 94 °C for 30 s, annealing at 60 °C for 30 s and extension at 72 °C for 10 mins, and final extension at 72 °C for 10 mins	^25,26^
	KPC-R	TTTTCAGAGCCTTACTGCCC	-		
*bla*_*NDM*_	NDM-F	GACAACGCATTGGCATAAG	447 bp		
	NDM-R	AAAGGAAAACTTGATGGAATTG	-		
*bla*_*VIM*_	VIM-F	ATTCCGGTCGGMGAGGTCCG	633 bp		
	VIM-R	GAGCAAGTCTAGACCGCCCG	-		
*mecA*	*mecA-*F	ATCGATGGTAAAGGTTGGC	530 bp	Initial denaturation at 94 °C for 4 mins followed by 30 cycles of denaturation at 94 °C for 30 s, annealing at 53 °C for 30 s and extension at 72 °C for 1 min with a final extension at 72 °C for 4 minutes.	^27^
	*mecA-*R	AGTTCTGCAGTACCGGATTTGC	-		


bp, base pairs.

### Statistical analysis

Data analysis was performed with Statistical Package for Social Sciences version 20 (SPSS Inc., Chicago, Illinois, United States). Comparison of mean values was done using the Student’s *t*-test for continuous variables and the chi-square test for categorical variables. Risk factors for infection of diabetic foot by MDR organisms were identified by logistic regression analysis. Logistic regression was used to determine predictive associations of variables that showed statistical significance by bivariate analysis. A *p*-value of less than 0.05 was considered to be statistically significant.

## Results

### Sociodemographic and clinical characteristics of patients

Ninety patients (53 male and 37 female) presented with 93 IDFUs during the 11-month study period. The patients ranged between 18 and 85 years (mean 54.7 ± 12.8 years) of age. Of the 93 cases of foot infections, 56 (60.2%) had lasted for at least 1 month, and 70 (75.3%) were in-hospital patients. Sixty-six (71.0%) of the ulcers were categorised as Wagner’s grade 3 and above. Most (*n* = 74; 82.2%) of the patients used antibiotics in the month preceding presentation at the healthcare facilities, while 93.3% (*n* = 84) had commenced antibiotics before collection of wound samples (Table 2).

**TABLE 2:** Association between clinical and sociodemographic variables of infected diabetic foot ulcer patients from tertiary healthcare facilities in Osun state, Nigeria, July 2016 to April 2017.

Variables	Multidrug-resistant bacteria				Bivariate analysis			Logistic regression analysis		
	Present		Absent		Crude odds ratio	95% confidence interval	*p*	Adjusted odds ratio	95% confidence interval	*p*
	*n*	%	*n*	%						
**Type of patients**										
Inpatient	58	82.9	12	17.1	2.60	0.88–7.72	0.08	-	-	-
Outpatient	13	65.0	7	35.0	-	-	-	-	-	-
**Age group (years)**										
< 30	4	80.0	1	20.0	0.78	0.304–1.987	0.69	-	-	-
30–49	16	84.2	3	15.8	-	-	-	-	-	-
50–69	44	77.2	13	22.8	-	-	-	-	-	-
70–79	5	71.4	2	28.6	-	-	-	-	-	-
80 and above	2	100.0	0	0.0	-	-	-	-	-	-
**Gender**										
Male	41	77.4	12	22.6	0.80	0.28–2.24	0.67	-	-	-
Female	30	81.1	7	18.9	-	-	-	-	-	-
**Type of diabetes**										
Type I	9	90.0	1	10	2.61	0.34–19.86	0.36	-	-	-
Type II	62	77.5	18	22.5	-	-	-	-	-	-
**Level of education**										
None	14	82.4	3	17.6	0.902	0.380–2.139	0.59	-	-	-
Primary	13	72.2	5	27.8	-	-	-	-	-	-
Secondary	25	78.1	7	21.9	-	-	-	-	-	-
Tertiary	19	82.6	4	17.4	-	-	-	-	-	-
**History of smoking**										
Yes	6	85.7	1	14.3	1.59	0.21–12.06	0.68	-	-	-
**Peripheral neuropathy**										
Yes	68	82.9	14	17.1	4.47	1.14–14.38	0.03	4.05	1.08–15.13	0.04*
**Evidence of systemic infection**										
Yes	37	82.2	8	17.8	1.50	0.54–4.11	0.44	-	-	-
**Ulcer grade (*N* = 93)**										
II	21	77.8	6	22.2	0.795	0.323–1.953	0.27	-	-	-
III	32	82.1	7	17.9	-	-	-	-	-	-
IV	18	85.7	3	14.3	-	-	-	-	-	-
V	3	50.0	3	50.0	-	-	-	-	-	-
**Foot infection duration (*N* = 93)**										
≤ 1 month	35	94.6	2	5.4	8.05	1.68–34.69	0.003	7.63	1.64–35.39	0.02*
> 1 month	39	69.6	17	30.4	-	-	-	-	-	-
**Previous admission for same ulcer**										
Yes	14	82.4	3	17.6	1.31	0.35–4.95	0.70	-	-	-
**Glycated haemoglobin at presentation (*N* = 51)**										
Poor	23	76.7	7	23.3	0.704	0.178–2.790	0.87	-	-	-
Fair	11	78.6	3	21.4	-	-	-	-	-	-
Good	6	85.7	1	14.3	-	-	-	-	-	-
**Admission duration (*N* = 70)**										
> 1 month	30	73.2	11	26.8	0.10	0.01–0.78	0.01	1.14	0.01–0.80	0.07
≤ 1 month	28	96.6	1	3.4	-	-	-	-	-	-
**Antibiotic use in the last 1 month before presentation**										
Yes	59	79.7	15	20.3	1.31	0.38–4.49	0.67	-	-	-
**Antibiotic use before sampling**										
Yes	67	79.8	17	20.2	1.97	0.38–10.13	0.45	-	-	-
**Poor treatment outcome of inpatients† (major amputations‡ or deaths) (*N* = 70)**										
Yes	31	93.9	2	6.1	5.74	1.18–27.86	0.02	5.11	1.23–29.67	0.03*

Note: *p*-values in bold are statistically significant by bivariate and multivariate (logistic regression) analyses.

*N* = 90 unless otherwise stated.

* , Statistical significance by binary logistic regression.

† , Inpatients were followed up during admission.

‡ , Major amputation: below knee amputation and above knee amputations.

### Predictors and treatment outcomes of multidrug-resistant bacterial infections in infected diabetic foot ulcer

Significant factors associated with the presence of MDR organisms in diabetic foot infections included peripheral sensory neuropathy, a foot infection duration of more than a month and admission duration of more than a month (Table 2). Further analysis with logistic regression however identified only peripheral neuropathy (adjusted odds ratio = 4.05, 95% CI: 1.08–15.13) and foot infection duration of more than a month (adjusted odds ratio = 7.63, CI: 1.64–35.39) as the predisposing factors for acquisition of multidrug-resistant bacteria among patients with IDFU. A substantial proportion (33/70; 47.1%) of the inpatients had poor treatment outcomes; poor outcomes noted in 53.4% (31/58) of patients with MDR infections included major limb amputation (below and above knee amputation) (18/58; 31.0%) and death (13/58; 22.4%). Infections by these MDR bacteria have significant association with poor treatment outcomes (adjusted odds ratio = 5.11, 95% CI: 1.23–29.67).

### Distribution of isolates among diabetic foot cases

All 93 wound specimens obtained from the 90 patients (three patients had bilateral foot ulcers) in the three healthcare facilities were positive on bacterial culture with only 10 (10.8%) of them yielding a single organism each. Among the polymicrobial cultures, 45 (48.4%) yielded two organisms per culture, 34 (36.6%) yielded three organisms per culture while 4 (4.3%) yielded four organisms per culture. Results further showed that there was a total of 218 organisms isolated from the 93 specimens cultured with an average of 2.34 organisms per sample. Of the organisms, 129 (59.2%) were Gram-negative aerobic and facultative anaerobic bacilli, 59 (27.1%) were Gram-positive aerobic cocci and 29 (13.3%) were anaerobic bacteria; only 1 (0.5%) was yeast. S. *aureus* (34; 15.6%) was the single most common organism followed by *E. coli* (23; 10.6%) and *Pseudomonas aeruginosa* (20; 9.2%). Others included *Klebsiella* spp. (19; 8.7%), *Citrobacter* spp. (19; 8.7%), *Enterococcus* spp. (14; 6.4%), *Enterobacter* spp. (11; 5.0%), *Proteus mirabilis* (10; 4.6%) and *Acinetobacter* spp (9; 4.1%). On the other hand, the predominant anaerobic bacteria were *Bacteroides* spp. (7; 3.2%) and *P. anaerobius* (6; 2.8%) as shown in Table 3.

**TABLE 3:** Bacterial aetiological agents of infected diabetic foot ulcers in tertiary healthcare facilities in Osun state, Nigeria, July 2016 to April 2017.

Organism categories	Organism name	*n*	%
Aerobic and facultative anaerobic Gram-positive cocci		59	27.1
	*Staphylococcus aureus*	34	15.6
	*Enterococcus faecalis*	10	4.6
	CoNS	4	1.8
	*Enterococcus mundtii*	2	0.9
	*Enterococcus faecium*	1	0.5
	*Enterococcus avium*	1	0.5
	*Streptococcus bovis*	1	0.5
	*Aerococcus* species	1	0.5
	Other CoPS¶¶	5	2.3
Aerobic and facultative anaerobic Gram-negative bacilli		129	59.2
	*Escherichia coli*	23	10.6
	*Pseudomonas aeruginosa*	20	9.2
	*Klebsiella* species†	19	8.7
	*Citrobacter* species‡	19	8.7
	*Enterobacter* species¶	11	5.0
	*Proteus mirabilis*	10	4.6
	*Acinetobacter* species§	9	4.1
	*Morganella morganii*	7	3.2
	*Hafnia alvei*	5	2.3
	*Providencia* species††	3	1.4
	*Salmonella enterica subsp.* Arizonae	2	0.9
	*Stenotrophomonas maltophilia*	1	0.5
Anaerobes		29	13.3
	*Bacteroides* species‡‡	7	3.2
	*Peptostreptococcus anaerobius*	6	2.8
	*Staphylococcus saccharolyticus*	4	1.8
	*Micromonas micros*	3	1.4
	*Prevotella melaninogenicus*	3	1.4
	*Lactobacillus acidophilus*§§	2	0.9
	*Streptococcus intermedius*§§	1	0.5
	*Fusobacterium varium*	1	0.5
	*Anaerococcus hydrogenalis*	1	0.5
	*Porphyromonas asaccharolyticus*	1	0.5
Yeast	*Candida albicans*	1	0.5

*N* = 218.

† , *Klebsiella* spp. (*K. pneumonae*-12; *K. oxytoca*- 7).

‡ , *Citrobacter* species (*C. freundii*- 8; *C. koserii*- 8; *C. sedlakii*- 3).

¶ , *Enterobacter* species (*E. aerogenes*- 7; *E. cloacae*- 4).

§ , *Acinetobacter* species (*A. baumannii*- 6; *A. johnsonnii*- 3).

†† , *Providencia* spp. (*P. alcalifaciens*- 2; *P. stuartii*- 1).

‡‡ , *Bacteroides* spp. (*B. fragilis* – 6; *B. vulgatus*- 1), CoNS- coagulase-negative *Staphylococcus* spp.

¶¶ , Other CoPS- coagulase-positive *Staphylococcus* spp. other than *S. aureus*.

§§ , Some of the strains are not strict anaerobes.

### Antibiotic resistance pattern of aerobic isolates

Gram-positive bacteria were highly resistant to trimethoprim-sulfamethoxazole (69.5%), penicillin G (66.1%) and gentamicin (40.1%) but demonstrated low-level resistance to piperacillin/tazobactam (6.8%) and amikacin (10.2%). On the other hand, Gram-negative bacteria were highly resistant to the third-generation cephalosporins which included ceftriaxone (56%), cefotaxime (55%) and ceftazidime (48.1%), as well as trimethoprim-sulfamethoxazole (89%), gentamicin (54.3%) and ciprofloxacin (54.3%). Low rates of resistance were however shown to ertapenem (6.4%), piperacillin/tazobactam (9.3%) and amikacin (12.4%).

### Prevalence and pattern of multidrug resistance among bacterial isolates in infected diabetic foot ulcer

Further analysis of resistance profiles in the organisms showed that of the 188 aerobic isolates, 121 (64.4%) were MDR, being resistant to one or more agents in at least three antibiotic classes (Table 4). The prevalence rates of MDR among GPC and GNB were 55.9% and 68.2%. Multidrug resistance rates were generally high among the isolated bacteria especially *Acinetobacter* species (88.9%), *Enterococcus* species (84.6%), *Enterobacter* species (81.8%) and *Citrobacter* species (73.7%). Overall prevalence of MDR bacteria among the IDFU cases was 80% (*n* = 74) with rates among in-patient and outpatient cases being 82.9% (*n* = 58) and 69.6% (*n* = 16). Twelve (35.3%) *S. aureus* were methicillin-resistant, while of the 129 GNB, 43 (33.3%) were ESBL-producing and 10 (7.8%) were carbapenem-resistant (Table 5). High ESBL production rates were seen among *Enterobacter* species (54.6%), *Klebsiella* species (52.6%), *Citrobacter* species (52.6%) and *E. coli* (43.5%). Other ESBL-producing species found were *Hafnia alvei* (1; 20.0%), *Providencia* spp. (1; 33.0%) and *Morganella morganii* (2; 28.6%). AmpC β-lactamase production was detected among *Citrobacte*r spp. (1; 5.3%), *E. coli* (2; 8.7%) and *M. morganii* (1; 14.3%). Carbapenem resistance was seen among *Acinetobacter baumannii* (4; 44.4%), *H. alvei* (2; 40.0%), *P. aeruginosa* (3; 15.0%) and *M. morganii* (1; 14.3%). Further tests showed that among the 10 carbapenem-resistant isolates, only four, *H. alvei* (2/4) and *A. baumannii* (2/4), were carbapenemase-producing.

**TABLE 4:** Prevalence of multidrug resistance among bacterial isolates from infected diabetic foot ulcer patients from tertiary healthcare facilities in Osun state, Nigeria, July 2016 to April 2017.

Bacteria isolates (*N*)	Multidrug-resistant isolates	
	*n*	%
**Gram-positive cocci (59)**	33	55.9
*Staphylococcus aureus* (34)	18	52.9
*Enterococcus* spp. (14)	11	78.6
Other† (5)	1	20.0
Coagulase-negative *Staphylococcus* (4)	1	25.0
*Streptococcus bovis* (1)	1	100.0
*Aerococcus* spp. (1)	1	100.0
**Gram-negative bacilli (129)**	88	68.2
*Escherichia coli* (23)	16	69.6
*Pseudomonas aeruginosa* (20)	10	50.0
*Klebsiella* species (19)	13	68.4
*Citrobacter* species (19)	14	73.7
*Enterobacter* species (11)	9	81.8
*Proteus mirabilis* (10)	6	60.0
*Acinetobacter* species (9)	8	88.9
*Morganella morganii* (7)	4	57.1
*Hafnia alvei* (5)	4	80.0
*Providencia* species (3)	2	66.7
*Salmonella enterica subsp.* Arizonae (2)	1	50.0
*Stenotrophomonas maltophilia* (1)	1	100.0
**Total (*N* = 188)**	**121**	**64.4**

† , Coagulase-positive *Staphylococcus* spp. other than *S. aureus*.

**TABLE 5:** Types and mechanisms of antibiotic resistance among bacterial agents of infected diabetic foot ulcer from tertiary healthcare facilities in Osun state, Nigeria, July 2016 to April 2017.

Bacteria isolates	MRSA/MRCoNS		VRSA/VRE		ESBL		AmpC		Carbapenem resistance	
	*n*	%	*n*	%	*n*	%	*n*	%	*n*	%
**Gram-positive cocci (59)**										
Staphylococcus *aureus* (34)	12	35.3	0	0.0	NA	NA	NA	NA	NA	NA
*Enterococcus* spp. (14)	NA	NA	0	0.0	NA	NA	NA	NA	NA	NA
CoNS (4)	1	25.0	NA	NA	NA	NA	NA	NA	NA	NA
Other† (7)	NA	NA	NA	NA	NA	NA	NA	NA	NA	NA
**Gram-negative bacilli (129)**	NA	NA	NA	NA	43	33.3	4	3.1	10	7.8
*Escherichia coli* (23)	NA	NA	NA	NA	10	43.5	2	8.7	0	0.0
*Klebsiella* spp. (19)	NA	NA	NA	NA	10	52.6	0	0.0	0	0.0
*Citrobacter* spp. (19)	NA	NA	NA	NA	10	52.6	1	5.3	0	0.0
*Enterobacter* spp. (11)	NA	NA	NA	NA	6	54.5	0	0.0	0	0.0
*Pseudomonas aeruginosa* (20)	NA	NA	NA	NA	0	0.0	0	0.0	3	15.0
*Proteus mirabilis* (10)	NA	NA	NA	NA	2	20.0	0	0.0	0	0.0
*Acinetobacter* spp. (9)	NA	NA	NA	NA	0	0.0	0	0.0	4‡	44.4
*M. morganii* (7)	NA	NA	NA	NA	2	28.6	1	41.3	1	14.3
*Hafnia alvei* (5)	NA	NA	NA	NA	1	20.0	0	0.0	2	40.0
*Stenotrophomonas maltophilia* (1)	NA	NA	NA	NA	1	100.0	0	0.0	0	0.0
*Providencia* spp (3)	NA	NA	NA	NA	1	33.3	0	0.0	0	0.0
*Salmonella enterica* subsp. Arizonae (2)	NA	NA	NA	NA	0	0.0	0	0.0	0	0.0

† , Other Gram-positive cocci included *Streptococcus bovis, Aerococcus* species and coagulase-positive *Staphylococcus* spp other than *S. aureus*.

‡ , These four species were *A. baumannii*.

MRSA, methicillin-resistant *Staphylococcus aureus*; MRCoNS, methicillin-resistant coagulase-negative *Staphylococcus spp*; VRSA, vancomycin-resistant *Staphylococcus aureus*; VRE, vancomycinresistant *Enterococcus*; ESBL, extended-spectrum β-lactamase; AmpC, AmpC β-lactamase; NA, not applicable.

### Detection of resistance genes

Ten (83.3%) of the 12 MRSA isolates harboured the *mec*A gene. At least one of the ESBL genes investigated was detected in 86.0% (*n* = 37) of the 43 ESBL-producing organisms. The most common ESBL gene detected was *bla*_CTX-M,_ harboured by 30 (69.8%) of the phenotypically confirmed ESBL-producing isolates. Others were *bla*_TEM_ (27; 62.8%) and *bla*_SHV_ (8; 18.6%) (Table 6). Thirty-one (72.1%) of the ESBL-producing GNB had at least two ESBL genes. Four of the 10 carbapenem-resistant species were phenotypically confirmed to be carbapenemase-producers. Carbapenemase and metallo-beta-lactamase genes were detected in all of the four phenotypically confirmed carbapenemase-producers; they were *bla*_VIM_ (*n* = 3), *bla*_KPC_ (*n* = 2) and *bla*_NDM_ (*n* = 1). These genes were detected in all of the carbapenemase-producing *H. alvei* (2/2) and *A. baumannii* (2/2).

**TABLE 6:** Detection of extended-spectrum beta-lactamase genes among phenotypically confirmed extended-spectrum beta-lactamase producing isolates from tertiary healthcare facilities in Osun state, Nigeria, July 2016 to April 2017.

Isolates with phenotypically confirmed ESBL (*N*)	Isolates with ≥ 1 ESBL genes		Prevalence of ESBL genes					
			CTX-M		TEM		SHV	
	*n*	%	*n*	%	*n*	%	*n*	%
*Escherichia coli* (10)	7	70.0	5	50.0	4	40.0	1	10.0
*Klebsiella* spp. (10)	8	80.0	7	70.0	6	60.0	3	30.0
*Citrobacter* spp. (10)	9	90.0	7	70.0	5	50.0	1	10.0
*Enterobacter* spp. (6)	6	100.0	6	100.0	5	83.3	2	33.3
*Morganella morganii* (2)	2	100.0	2	100.0	2	100.0	0	0.0
*Proteus mirabilis* (2)	2	100.0	1	50.0	2	100.0	0	0.0
*Hafnia alvei* (1)	1	100.0	1	100.0	1	100.0	1	100.0
*Stenotrophomonas maltophilia* (1)	1	100.0	1	100.0	1	100.0	0	0.0
*Providencia alcalifaciens* (1)	1	100.0	0	0.0	1	100.0	0	0.0
**Total (43)**	**37**	**86.0**	**30**	**69.8**	**27**	**62.8**	**8**	**18.6**

ESBL, extended-spectrum β-lactamase.

## Discussion

This study shows that a wide range of bacteria are agents of infection of diabetic foot ulcers; it also reveals the high level of antibiotic resistance among the aerobic and facultative anaerobic bacteria with a large proportion of patients having multidrug-resistant infection leading to poor treatment outcomes. Infected diabetic ulcers continue to be polymicrobial infections involving aerobic as well as obligate anaerobic organisms. Infected diabetic foot ulcers in this study have an average of two different bacteria implicated in the disease, and this is typical of diabetic foot infections across sub-Saharan Africa and Asia.^29^ Gram-negative aerobic bacteria including *E. coli, P. aeruginosa, Klebsiella* species and *Enterobacter* species predominate, reflecting the long-standing nature of these infections as a consequence of poor health-seeking behaviour in low-resourced developing countries.^8,30^ Gram-negative bacteria are more commonly implicated in infected diabetic ulcers in developing countries where most patients present late to healthcare facilities with advanced diseases.^29^ Furthermore, a wide range of anaerobic bacteria primarily *Bacteroides* species and *P. anaerobius* are important agents of the infections and were isolated from a third of the cases in this study. This suggests infections that are chronic and below the superficial layers of the skin.^31^ Anaerobic bacteria account for 13.3% of the organisms isolated in this study, a higher prevalence than previously reported in the institution^32^ and may be attributable to deployment of a better anaerobic culture method in which specimens were processed and incubated in a Bactron anaerobic chamber. Higher prevalence rates of obligate anaerobes were however reported by Ikeh et al. in Jos, Nigeria (17%),^33^ and Al-Benwan in Kuwait (15.3%).^34^ Low rates noted by Richard et al. (1%)^35^ in France and Yates et al. (1%)^36^ in Australia may be due to the fact that most patients tend to seek medical care early enough in countries with good health insurance coverage which will enable a higher proportion to present with low-grade foot infection.^37^

Antibiotic resistance remains a huge problem among diabetic foot ulcer infections; it worsens prognosis and makes treatment outcomes poor.^38^ Multidrug-resistant bacteria were common (74/93; 80%) among IDFU cases in this study, and this is possibly due to inappropriate antibiotics use and unrestricted access to antimicrobial drugs in many low- and middle-income countries.^39^ This is similar to findings in studies conducted in other developing countries^40,41^ but contrasts findings of several studies in high-income countries including France which reported low prevalence of MDR bacteria among patients with IDFU.^38,42^ A wide spectrum of aerobic bacteria isolated in this study were found to be multidrug-resistant, comparable to findings elsewhere in Africa and Asia with high MDR rates involving mainly *S. aureus, Enterobacteriaceae*, and *P. aeruginosa*.^41,43^

One in every three isolates of *S. aureus* in this study was MRSA. Although prevalence of MRSA appears to be rising in Africa, most of the countries have rates lower than 50%.^44^ Our study also revealed that *mecA*, the most common determinant that confers methicillin resistance on *S. aureus*, was detected in 83.3% of the MRSA strains and this is similar to the observation of Chaudhry et al. who detected the gene in 20 (84%) of the 25 phenotypically confirmed MRSA isolates.^45^ MRSA strains that lack the *mec*A gene may demonstrate methicillin resistance on account of alternate mechanisms of penicillin resistance such as the possession of *mecC,* a variant of *mecA* discovered in 2011, or other mutations in genes encoding penicillin-binding proteins.^46^

Extended-spectrum β-lactamases, which confer resistance to expanded-spectrum cephalosporins, were produced by 33.3% of all GNB isolated; all but two of the ESBL-producing GNB belonged to the family *Enterobacteriaceae* and included *E. coli, Klebsiella* and *Citrobacte*r species. The burden of ESBL-producing GNB is enormous among patients with IDFU especially in poor-resourced countries with prevalence rates being reported to range from 23% to 49% across Africa and Asia.^22,43,45,47,48^ The most prevalent ESBL gene was the CTX-M which has been reported as the most predominant variant worldwide.^49^ In the present study, only 10 (7.8%) of the Gram-negative bacteria were resistant to the carbapenems. Carbapenem resistance-determining genes were present in *A. baumannii, H. alvei* and *M. morganii*. Carbapenems as drugs of last resort in the treatment of resistant GNB infections have variable but increasing rates of resistance.^13^

Independent risk factors for acquisition of MDR bacteria found in our study are peripheral sensory neuropathy and foot infection duration longer than a month. Peripheral neuropathy does not only make diabetics susceptible to foot ulceration but also makes insensate (neuropathic) foot ulcers become more extensive due to continuous painless trauma. Loss of protective pains could cause patients not to present to healthcare facilities early enough. In developing countries, such patients with more chronic infections (> 1 month duration) would have engaged in self-prescribed antibiotic use for a prolonged period of time leading to selective pressure and emergence of MDR foot infection.^39,50^ This is similar to reports among IDFU cases from India.^40,51,52^ Other authors also documented the prolonged duration of wound infection as a predictor of infection of diabetic foot ulcers with MDR bacteria.^53,54^ Contrary findings have however been documented from other studies in China, Iran and Portugal.^41,42,55^ Our finding is also discordant with the report of Noor et al. which established that ulcer size is a risk factor for infection by MDR organisms.^54^ This study also observed a significant association between presence of MDR bacteria in IDFU and long duration of hospitalisation (> 1 month) similar to previously documented reports by another author in Turkey.^14^ We did not find any sociodemographic factors that were significantly associated with the occurrence of MDR IDFU in our study in agreement with other reports.^40,52,53^ In contrast, Trivedi et al. in the United States noted smoking as an independent risk factor for multidrug-resistant foot wound infection.^56^ Furthermore, in this study, infection of diabetic foot ulcers by MDR pathogens was found to have a significant association with poor treatment outcomes including major limb amputation and mortality. In agreement with our findings, the adverse effects of MDR diabetic foot infection on treatment had been underscored in a systematic review and meta-analysis of data from 28 studies reporting a treatment failure rate of 22.7% and significant association between MDR foot infections and treatment failure.^57^

### Limitations

The limitation of the study was that the number of patients recruited was limited to 90 and this was because the study was time-bound. Also, outpatients could not be followed up because of the multicentre nature of the study. Resistance profiles of obligate anaerobic bacteria could not be determined and whole genome sequencing (for strain relatedness) was also not done due to lack of financial support for the study.

### Conclusion

The spectrum of agents causing IDFU is wide and includes numerous species of aerobic and anaerobic bacteria. There is a high prevalence of MDR aerobic bacteria among them which poses a great limitation to the effective treatment of cases.
